# Image Artifacts in Concurrent Transcranial Magnetic Stimulation (TMS) and fMRI Caused by Leakage Currents: Modeling and Compensation

**DOI:** 10.1002/jmri.21749

**Published:** 2009-05

**Authors:** Nikolaus Weiskopf, Oliver Josephs, Christian C Ruff, Felix Blankenburg, Eric Featherstone, Anthony Thomas, Sven Bestmann, Jon Driver, Ralf Deichmann

**Affiliations:** 1 Wellcome Trust Centre for Neuroimaging, UCL Institute of Neurology, University College LondonLondon, United Kingdom; 2 UCL Institute of Cognitive Neuroscience, University College LondonLondon, United Kingdom; 3 Department of Neurology and Bernstein Center for Computational NeuroscienceCharité, Berlin, Germany; 4 The Magstim Company LimitedWhitland, Wales, United Kingdom; 5 Sobell Department of Motor Neuroscience and Movement Disorders, UCL Institute of Neurology, University College LondonLondon, United Kingdom; 6 University Hospital, Brain Imaging CenterFrankfurt, Germany

**Keywords:** transcranial magnetic stimulation, TMS, functional magnetic resonance imaging, fMRI, MR artifacts, leakage current

## Abstract

**Purpose:**

To characterize and eliminate a new type of image artifact in concurrent transcranial magnetic stimulation and functional MRI (TMS-fMRI) caused by small leakage currents originating from the high-voltage capacitors in the TMS stimulator system.

**Materials and Methods:**

The artifacts in echo-planar images (EPI) caused by leakage currents were characterized and quantified in numerical simulations and phantom studies with different phantom-coil geometries. A relay-diode combination was devised and inserted in the TMS circuit that shorts the leakage current. Its effectiveness for artifact reduction was assessed in a phantom scan resembling a realistic TMS-fMRI experiment.

**Results:**

The leakage-current-induced signal changes exhibited a multipolar spatial pattern and the maxima exceeded 1% at realistic coil-cortex distances. The relay-diode combination effectively reduced the artifact to a negligible level.

**Conclusion:**

The leakage-current artifacts potentially obscure effects of interest or lead to false-positives. Since the artifact depends on the experimental setup and design (eg, amplitude of the leakage current, coil orientation, paradigm, EPI parameters), we recommend its assessment for each experiment. The relay-diode combination can eliminate the artifacts if necessary. J. Magn. Reson. Imaging 2009;29:1211–1217. © 2009 Wiley-Liss, Inc.

COMBINING TRANSCRANIAL MAGNETIC STIMULATION (TMS) (1,2) with functional MRI (fMRI) (3) opens new perspectives, since the active manipulation of brain function can be combined with an accurate detection of activity throughout the brain. The technical feasibility of combined TMS and fMRI was originally demonstrated by Bohning et al (4) and followed by several technical refinements (5–8), while more recent studies have gone on to combine TMS-fMRI in a variety of applications (for a recent overview, see Ref. 9). Despite its increasing application, TMS-fMRI remains technically challenging. Moreover, the MRI environment poses additional safety issues in conjunction with TMS (4). Several studies (4–6) have reported image artifacts in gradient echo echo-planar imaging (GE EPI) caused by application of TMS in fMRI, and identified different artifact sources.

Here, we identify and investigate a novel type of image artifact that can arise during concurrent TMS-fMRI, and outline strategies for circumventing this. The image artifact is caused by magnetic field inhomogeneities due to small (residual) currents in the TMS coil, present even when no TMS pulse is applied. This is due to the following effect: In general, TMS stimulators make use of high-voltage capacitors for creating strong currents through the TMS coil when they are discharged. However, the high voltage can also drive a small leakage current even when the system is *not* being discharged, since the electronic switch that controls the discharging has a finite resistance. This leakage current through the TMS coil generates a local magnetic field distortion, and hence EPI geometric and intensity distortion (10). Here we model the new type of artifacts and also devise and validate a technique for dealing with them, ie, adding a relay-diode combination to the TMS stimulator circuit that effectively bypasses any leakage currents past the TMS coil (Fig. 1).

**Figure 1:**
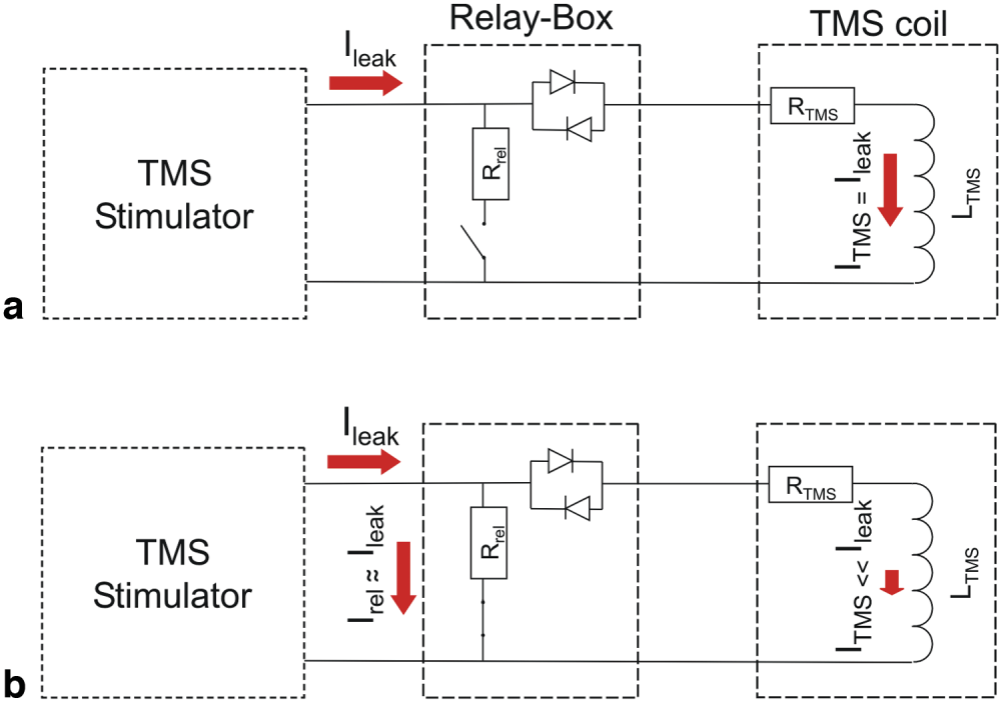
Minimizing leakage currents through the TMS coil. **a:** Low-frequency leakage currents I_leak_ are only limited by the low resistance R_TMS_ of the TMS coil. Therefore, even small residual voltages can cause significant leakage currents I_TMS_ flowing through the TMS coil. **b:** To minimize I_TMS_, a relay with minimal resistance R_rel_ is inserted in parallel to the TMS coil and two high-voltage diodes are inserted in series. The diode arrangement ensures that the effective coil resistance R_TMS_ is very large (>100 kΩ) when the voltage across the coil and diodes is less than ≈0.5 V. Thus, when the relay is closed it shorts the leakage current, preventing it from flowing through the TMS coil. [Color figure can be viewed in the online issue, which is available at www.interscience.wiley.com.]

## MATERIALS AND METHODS

### Modeling of EPI Artifacts Caused by Small Currents Through the TMS Coil

**Figure 2:**
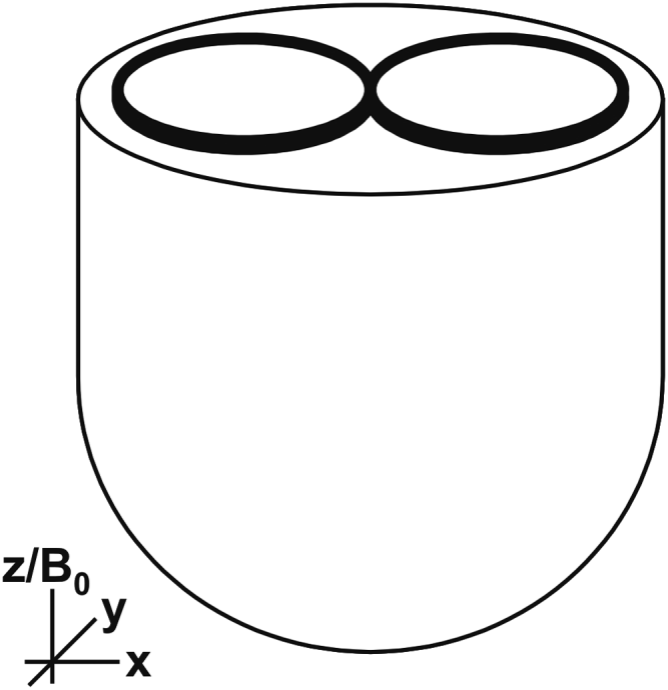
Geometry of the TMS-phantom setup used for simulations and experiments. The static magnetic field B_0_ and the slice select direction pointed in the z-direction. The PE direction was either in the y- or x-direction.

Any electric current I_TMS_ flowing through the TMS coil will generate a magnetic field 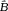
^*TMS*^∝*I*_*TMS*_. For small 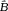
^*TMS*^ compared to the main field 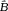
^0^, the strength of the net magnetic field can be approximated as ∥
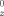
∥ ≈ *B*_*z*_^0^ + *B*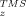 (main field in the z-direction; for a detailed derivation estimating all TMS field components, see, for example, Ref. 11). 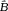
^*TMS*^ will cause geometric distortions (mainly) in the phase-encoding (PE) direction of the EPI image and concomitant signal changes due to compression/stretching of voxels (10). The magnetic field 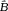
^*TMS*^ and intensity distortions were calculated for a simplified figure-of-eight TMS stimulation coil that approximated the custom-built TMS coil used at our site, modeled as two circular loops of wire. The field 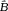
^*TMS*^ was determined using numerical integration of the Biot–Savart law and the field gradient by numerical differentiation of the field. Simulations were performed for the coil parallel to the x–y plane (=transverse, orthogonal to 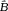
_0_) and the PE direction either in the x (left–right) or y (anterior–posterior) direction. The long axis of the coil (connecting the two loops) pointed in the x direction. Figure 2 illustrates the geometry of the simulated setup.

### Experiment 1: Phantom Measurements With Different TMS Coil Currents

EPI data at different currents *I*_*TMS*_ were acquired for the same actual TMS coil configurations as described above for the simulations. Data were acquired with a 1.5 T whole-body MRI scanner (Magnetom Sonata, Siemens Medical Solutions, Erlangen, Germany) using the standard CP head coil and body transmit coil. TMS was conducted using a MagStim Rapid system (Whitland, Wales, UK) with a custom-built MR-compatible figure-of-eight stimulation coil (MRI coil D70, S/N.15250; Magstim): two windings of 10 turns each, inner/outer diameter winding 53 mm/86 mm, thickness of TMS coil's casing (measured as distance between outer coil surface and center of winding) ≈15 mm, coil resistance R_TMS_ ≈100 mΩ, maximal voltage/current at 100% stimulator output ≈1.65 kV/5 kA. A dome-shaped water phantom was positioned with the phantom's flat surface parallel to the transverse plane (see Fig. 2). The TMS stimulation coil was fitted tangentially to the flat surface of the phantom.

The phantom was scanned with a single-shot EPI sequence using the following parameters: 60 slices, slice thickness = 2.5 mm, interslice gap = 1.25 mm, 64 × 96 matrix (ROxPE), field of view (FOV) = 250 × 250 mm^2^, FOV oversampling in PE direction = 50%, BW_PE_ = 20.833 Hz/Px, echo time TE = 42 ms, repetition time TR = 5400 ms, flip angle α = 20°. A controlled DC current was injected into the coil (*I*_*TMS*_ = [0.5, 1, 2, 3, 4, 5] mA). At *I*_*TMS*_ = 0 mA and *I*_*TMS*_ = 5 mA, double echo FLASH images (with parameters according to Ref. 12) were acquired for estimating field maps using the FieldMap toolbox for SPM5 (13,14).

To improve the signal-to-noise ratio (SNR), images were spatially smoothed with an isotropic Gaussian kernel of full-width at half-maximum (FWHM) = 8 mm. To estimate the change in image intensity due to the experimentally induced current flowing in the TMS coil, SPM5 linear regression analysis (Wellcome Trust Centre for Neuroimaging, UCL, London, UK) was performed on the images with *I*_*TMS*_ as the independent variable.

### Experiment 2: MR Artifacts Due to Leakage Currents That Depend on the TMS Stimulator Output Setting and Their Minimization by Use of a Relay-Diode Combination

In Experiment 1, controlled currents were driven through the TMS coil using an external power supply to estimate the level of current-related artifacts. In our second phantom experiment, we studied the more practically relevant case of actual leakage currents originating from the TMS stimulator itself (see Fig. 1). Since this leakage current was expected to depend on the TMS stimulator output level, the ensuing EPI artifacts were assessed by systematically varying the stimulator output settings.

The TMS setup was almost identical to that used in the previous phantom experiment. However in addition, a high-voltage relay (DAR70510, Crydom SSR, Dorset, UK) was introduced in parallel and a high-voltage diode (MDD95-16N1B, Ixys, Milpitas, CA) arrangement in series to the TMS coil (see Fig. 1; Magstim ES9486). The parallel arrangement of the diodes results in a high resistance (>100 kΩ) when the voltage across them is less than ≈0.5 V. Thus, when the bypass relay is closed, any leakage current flows primarily through the relay (I_rel_) and *not* through the TMS coil (I_TMS_). While the relay is closed or its status changed, TMS pulses must not be applied, since this type of high-voltage relay does not support the high currents (in the range of kA) of a TMS pulse. Therefore, the relay and TMS stimulator were controlled via a dedicated DOMINO 1 microcontroller (Micromint, Lake Mary, FL).

The positions of the TMS coil and phantom were comparable to Experiment 1 (see Fig. 2) and the EPI parameters were identical to the previous Experiment 1, except for the reduced number of slices per image volume of 20, TR = 1800 ms, and flip angle α = 30° (PE direction along y). In a single experimental run, 1205 image volumes were recorded while the TMS stimulator was switched between different output levels remotely (0%, 25%, 50%, 75%, or 99%) in a parametric block design. Each block started with 10 image volumes during which the stimulator was set to 50% output, followed by 10 image volumes where one of the other four intensities was randomly selected. A total of 15 blocks was presented for each of the four intensity level combinations. No TMS pulses were applied during the experiment.

All image processing steps were performed with SPM5. After offline image reconstruction (15) and removal of the first 5 volumes of the time series, images were realigned and spatially smoothed using an isotropic Gaussian kernel with 8 mm FWHM. The voxel-wise time-series were then highpass-filtered (128 sec cutoff) and regressed on a composite general linear model (GLM) with two regressors, representing the constant session mean and the scan-wise output level of the TMS stimulator. The percent signal change for the maximal stimulator output of 100% (note that only 99% could be achieved with our setup) was estimated from the fitted GLM. To assess the effectiveness of the artifact reduction method, the experiment was run twice: once with the relay open and once with the relay closed.

## RESULTS

### Simulation and Experiment 1: EPI Artifacts Due to Currents Through the TMS Coil

**Figure 3:**
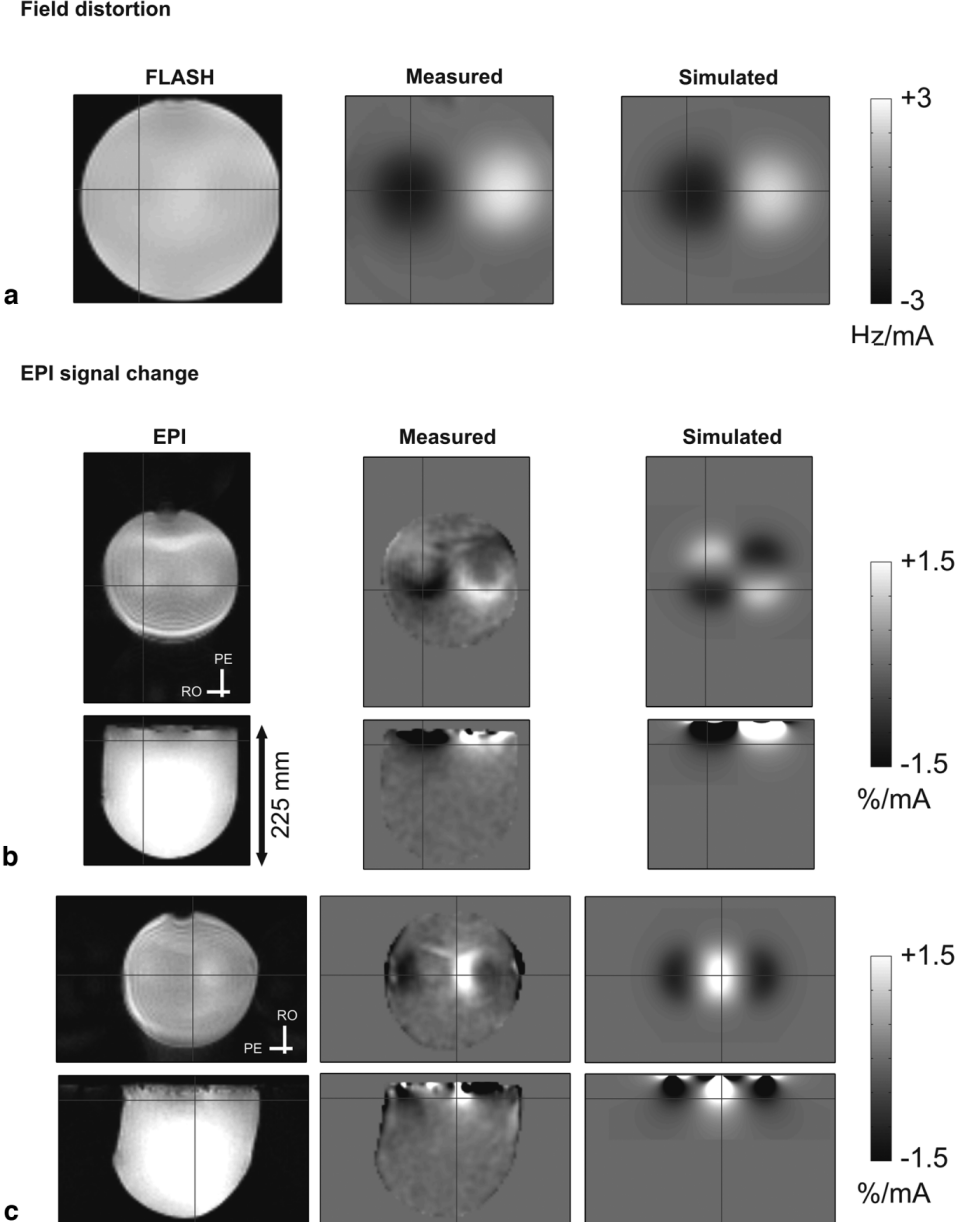
Magnetic field changes and EPI intensity distortions caused by currents flowing through the TMS stimulation coil. Illustrative transverse slice ≈4 cm away from the center of the coil. **a:** Magnetic field changes expressed as frequency offsets (Hz) per mA current. FLASH magnitude image (left), plus measured (center), and simulated (right) frequency offset. **b:** Relative EPI signal changes per mA current applied to the TMS coil when the phase-encoding (PE) direction was anterior-posterior. EPI magnitude image (left), measured (center) and simulated (right) signal change. The bottom row shows coronal slices to appreciate how the level of artifacts decreased with increased distance from the TMS coil. **c:** Same as b, but the PE direction was left–right. An imperfect shim due to the nonspherical phantom geometry led to the clearly visible distortion in the EPI magnitude image (bottom row).

Figure 3 shows the results of the simulation of the magnetic field and resulting signal distortion in EPI for an illustrative transverse slice ≈4 cm from the center of the TMS coil (estimated from the distance between the slice and the phantom's edge + 1.5 cm for the plastic case of the TMS coil)—a realistic distance between the TMS coil and stimulated cortical areas in concurrent TMS-fMRI experiments in humans.

For the anterior–posterior PE direction, maximal intensity distortions of min/max = [−1.48, 1.34]% per 1 mA current were measured in the presented slice. The corresponding simulation predicted ±0.98%/mA. For the left–right PE direction [−1.27, 2.48]%/mA were measured, as compared to [−1.01, 1.98]%/mA derived by the simulation (edge artifacts excluded).

### Experiment 2: MR Artifacts Due to Leakage Currents That Depend on the TMS Stimulator Output Setting and Their Minimization by Use of a Relay-Diode Combination

**Figure 4:**
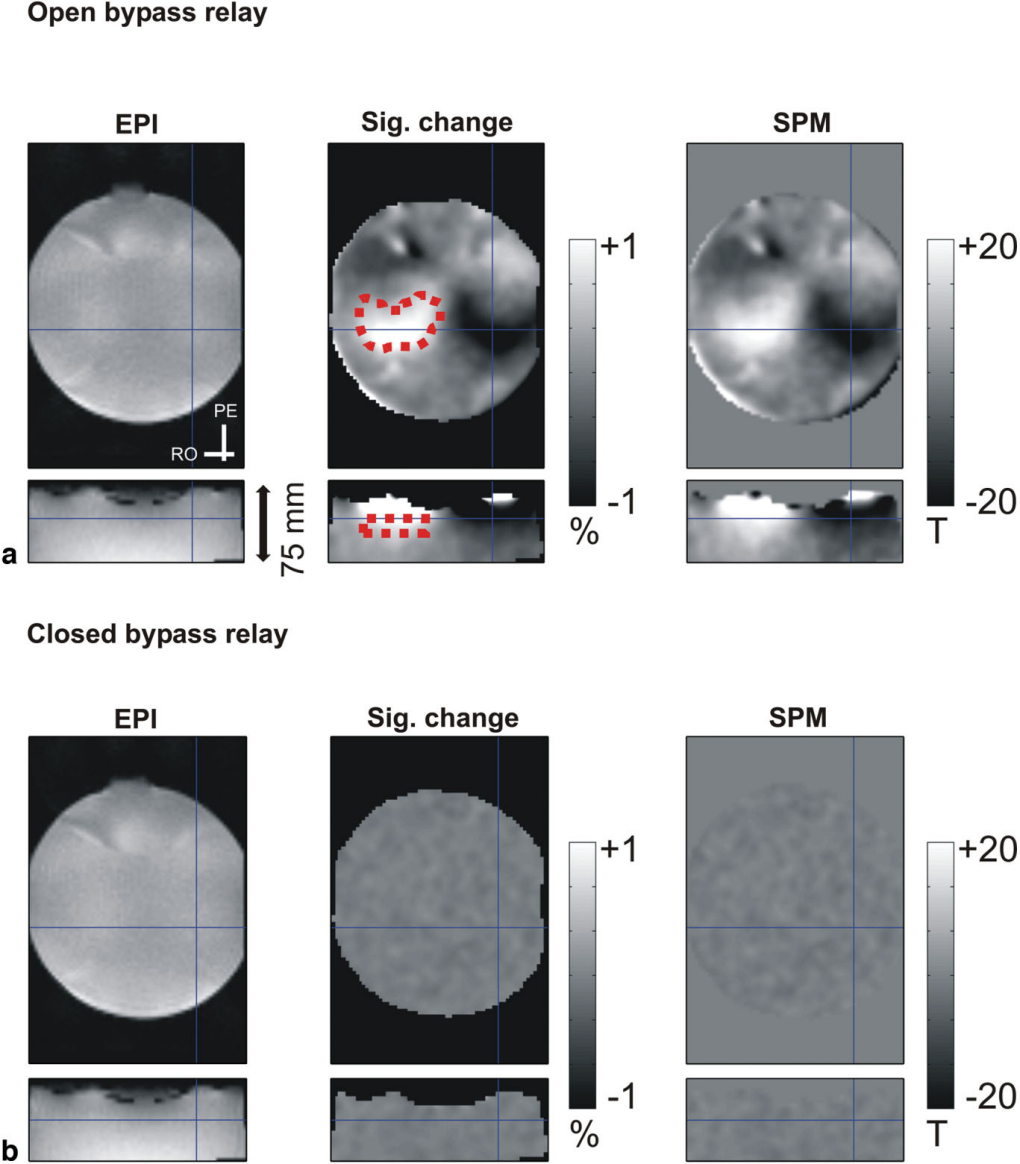
Effects of actual TMS stimulator leakage currents and elimination of these by a bypass relay and diodes: EPI magnitude image (left), percent signal change at maximal stimulator output setting (center), statistical parametric map (SPM, right). The SPM is a statistical t-map following a test for linear dependence of the signal intensity changes on the stimulator output setting. Systematic artifacts were observed only when the relay was open **(a)**, but were reduced below the noise level when the relay was closed **(b)**. The presented slice was ≈4 cm away from the TMS coil center. The red dotted outline delineates a region of interest for further analysis (for results, see Fig. 5). The bottom row shows coronal images to appreciate how the level of artifacts decreased with increased distance from the TMS coil.

**Figure 5:**
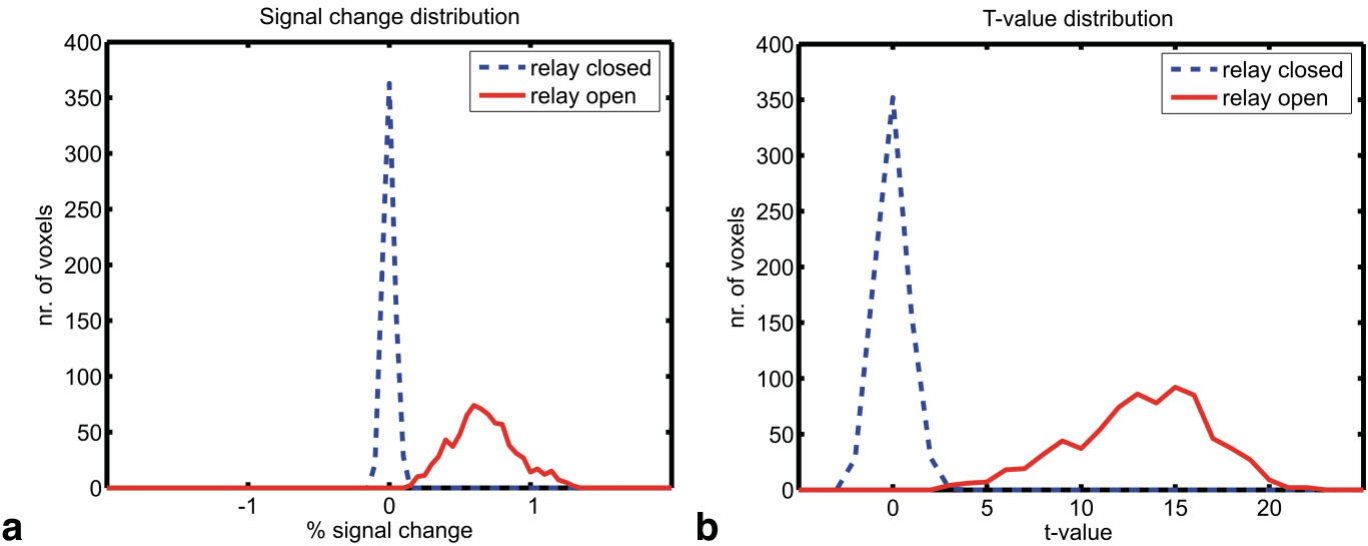
Effects of TMS stimulator leakage currents and compensation by a bypass relay and diodes: analysis of the region of interest (ROI) defined in Fig. 4a (center). Histograms of (**a**) signal changes at the maximal stimulator output setting and (**b**) corresponding statistical t-values are plotted for all voxels within the ROI. The shifted means for measurements when the relay was open (solid red) indicate significant artifacts due to leakage currents. The zero means for measurements when the relay was closed (dashed blue line) indicate successful elimination of leakage current effects. [Color figure can be viewed in the online issue, which is available at www.interscience.wiley.com.]

Figure 4a (center) shows how the EPI intensity distortions depended on the TMS stimulator output setting in Experiment 2 (4 cm away from TMS coil center). When the bypass relay was open, artifactual signal changes at the maximal stimulator output were larger than ±1% in certain areas underneath the TMS coil. The statistical t-map (Fig. 4a, right) shows that the measured signal intensity changes depended systematically on the stimulator output. When the same experiment was run with a closed bypass relay, the intensity distortions disappeared (Fig. 4b). This removal of the leakage current artifact by the closed bypass relay is further illustrated by analysis of percent signal change and t-values in a region of interest (ROI) underneath the coil (Fig. 5). Given the result from Experiment 1 that a current of 1 mA causes a maximal signal change of ≈1%–1.5%, the maximal value of ≈1% signal change found here indicates that the maximal leakage current caused by the TMS stimulator was on the order of 0.7–1 mA.

## DISCUSSION

We have identified a new type of image artifact that can occur in concurrent TMS-fMRI experiments. Leakage currents in the TMS stimulation coil distort the magnetic field inside the imaged object or brain, resulting in geometric distortion in the EPI phase-encoding (PE) direction. The geometric distortions lead to compression/stretching of the imaged voxels and subsequently to signal decreases/increases. As shown here, the TMS stimulator's high-voltage capacitors can be a source of charge-level-dependent leakage currents. Even small leakage currents of ≈1 mA as observed in our experiments can cause locally *maximal* EPI signal distortions on the order of 1% in a slice 4 cm away from the TMS coil (corresponding to a 2.5 cm distance from the outer plastic casing as measured orthogonally to the coil plane)—a realistic coil-cortex distance in TMS (16).

If the leakage currents were constant, they would only cause an offset in the signal amplitude. Such an offset would not affect the results of a conventional fMRI analysis, since it is only sensitive to signal changes over time (3). However, leakage currents depend on the capacitor charge, which some experimental designs systematically vary to achieve TMS pulses of different intensities (similar to Experiment 2). Furthermore, the capacitor charge will fluctuate slightly, since the charge slowly decays over time and is automatically replenished by the TMS stimulator, resulting in brief bursts of leakage current that are also eliminated by the relay-diode combination (not shown here). These spurious signal fluctuations can increase the noise level beneath the TMS coil, possibly masking blood oxygenation level-dependent (BOLD) responses. They can also result in false-positive inferences in fMRI. In particular, TMS-fMRI experiments studying the dependence of the BOLD response on TMS pulse intensity are susceptible, since the spurious signal changes would systematically depend on the stimulator output setting and can thus at least partially simulate BOLD response variations.

Here we have developed a method to minimize the leakage current flowing through the TMS coil by introducing a relay in parallel and diodes in series with the TMS coil (see Fig. 1). When the relay is closed, the leakage current flows primarily through the relay and not through the TMS coil, reducing the current through the coil by several orders of magnitude and suppressing the artifacts to well below the background noise level.

For brevity, we only presented data from experiments on a phantom without discharging the stimulator. However, the theory and the mechanism of the leakage currents indicate that these results readily generalize to human TMS-fMRI experiments. The effects do not depend on what object is imaged or whether TMS pulses are applied during an experiment. Further, the geometric setup used here is typical for human studies (eg, distance between TMS coil and imaging plane) and simulations show that other coil orientations result in artifacts of the same order of magnitude (not shown here). Most important, the relay-diode artifact suppression was successfully applied in human TMS-fMRI experiments (see, eg, Refs. 17,18).

We also note that the present solution of the relay-diode combination should only be added to the TMS stimulation circuit with considerable care. First, the relay does not sustain the high currents of a TMS stimulation pulse. We use a dedicated microcontroller to ensure that TMS pulses cannot be discharged when the relay is closed or its operating state is being changed. Second, the closed relay and diodes change the impedance of the TMS stimulation circuit. In particular, eddy-current artifacts may be exacerbated if the diode's on-voltage is small compared to the voltage induced by the imaging gradients (for reference, here we used maximal gradient slew rates of 127.8/69.6/72.7 mT/m/ms in the x/y/z direction), effectively shorting the circuit. Therefore, careful testing of possible interactions with the radiofrequency and gradient fields is advised (applying the tests described below and, eg, Ref. 19). Third, the relay has a limited lifetime and its functioning needs to be tested regularly.

We recommend the assessment of this potential artifact for each individual TMS-fMRI experiment. An established test method is to run the same experiment on a phantom as used later on the human subjects (see, for example, the supplementary material in Ref. 8), followed by careful analysis. Detection of potential artifacts is further facilitated by using a real-time image quality assurance system (20).

In conclusion, we have identified a new type of artifact potentially arising in concurrent TMS-fMRI experiments, caused by small leakage currents flowing through the stimulation coil. The addition of a bypass relay and diodes into the TMS circuit can efficiently eliminate any leakage current to improve image quality beneath the TMS coil, providing one useful step toward a more accurate assessment of BOLD activity close to the TMS stimulation site. Since the artifact depends on various factors, for optimal results we recommend assessment of this potential artifact for each individual TMS-fMRI experiment and application of the correction method presented here if necessary.
